# Acute abdomen revealed *Streptococcus gordonii* infective endocarditis with systemic embolism

**DOI:** 10.1093/omcr/omab145

**Published:** 2022-01-24

**Authors:** Chee Yik Chang, Yi Lung Gan, Anuradha P Radhakrishnan, Edmund L C Ong

**Affiliations:** Medical Department, Hospital Selayang, Selangor, Malaysia; Medical Department, Hospital Selayang, Selangor, Malaysia; Medical Department, Hospital Selayang, Selangor, Malaysia; Faculty of Medical Sciences, University of Newcastle Medical School, Newcastle upon Tyne, UK

## Abstract

Infective endocarditis can result in potentially fatal complications such as heart failure, systemic embolization, mycotic aneurysm and neurological complications. Staphylococci and streptococci are the most common causative agents of infective endocarditis, with *Streptococcus gordonii* being a rare cause. We present a case of infective endocarditis in a young patient who presented with an acute abdomen 2 months after being diagnosed with cerebrovascular accident. An abdominal computed tomography revealed superior mesenteric artery thrombosis, and infarct in the right kidney and spleen as a result of systemic septic embolism. Echocardiography showed numerous vegetations at the aortic and mitral valves. Infective endocarditis was diagnosed based on echocardiographic findings and positive blood cultures for *S. gordonii*. He was treated with intravenous benzylpenicillin and was also referred for surgical intervention.

## INTRODUCTION

Infective endocarditis is a potentially fatal condition with significant morbidity and mortality. Among the most common complications of infective endocarditis were heart failure, periannular abscess, systemic embolization, mycotic aneurysm and neurological complications [1].

Streptococci are the second most common cause of infective endocarditis after staphylococci. The viridans group streptococci (VGS) were found to be responsible for one-third of all streptococcal infective endocarditis cases, with *S. mitis* group streptococci being the most common [2].

*S. gordonii* infection can cause infective endocarditis, septic arthritis and spontaneous bacterial peritonitis [3]. Herein, we present a case of *Streptococcus gordonii* infective endocarditis complicated by systemic embolization to multiple sites.

## CASE PRESENTATION

A 28-year-old Bangladeshi man with no prior medical history initially presented with right-sided body weakness, and a brain computed tomography (CT) scan revealed left middle cerebral artery infarct. Due to financial constraints, he was treated with acetylsalicylic acid 150 mg daily and simvastatin 40 mg daily. Two months later, he presented to our hospital with 3 days of generalized, colicky abdominal pain and 1 week of fever. He had no diarrhea, vomiting, recent abdominal trauma or surgical history.

His initial vital parameters revealed a blood pressure of 119/50 mm Hg and a temperature of 38.2°C. He was also tachycardic but saturating well in ambient air. On general examination, there was a collapsing pulse and finger clubbing, but no other signs of infective endocarditis. An oral examination revealed dental plaques but no caries. A thorough cardiovascular examination revealed the presence of an early diastolic murmur over the Erb’s point and a pansystolic murmur over the apical region radiating to the left axillary area. There were no signs of heart failure. A neurological examination revealed residual weakness in the right upper and lower limbs, with power of 4/5 compared to the left side. An abdominal examination revealed generalized tenderness throughout the abdomen, with the umbilical region being the most tender.

**Figure 1:**
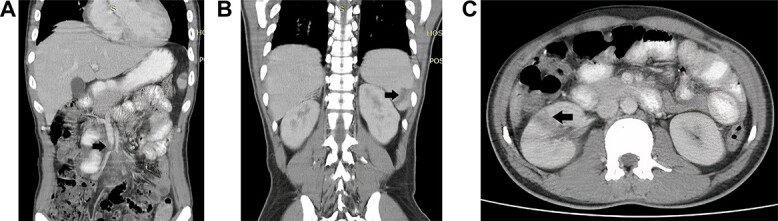
Computed tomography of the abdomen showing (a) thrombosis at the distal part of the superior mesenteric artery, (b) a wedge-shaped hypodense lesion in the lower pole of the spleen, (c) a hypodense lesion in the midpole of the right kidney.

The initial full blood count revealed a white cell count of 15 × 10^9^/L, hemoglobin of 8 g/dl, and normal platelet counts. The electrocardiogram revealed sinus tachycardia. The chest and abdominal radiographs were normal. The patient was referred to the surgical team for an acute abdomen with a provisional diagnosis of perforated appendix. As a result, an urgent abdominal CT was performed, which revealed superior mesenteric artery thrombosis as well as right renal and splenic hypodense lesions, indicating infarction from septic emboli (Fig. 1). There were no signs of a perforated appendix.

**Figure 2:**
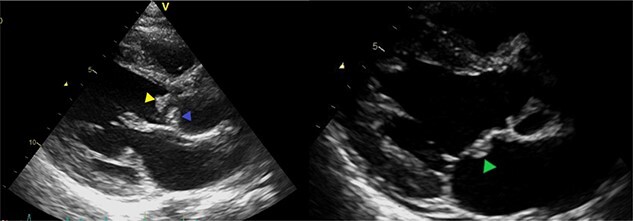
Echocardiography showing vegetations at the aortic and mitral valves (anterior aortic valve = yellow arrow; posterior aortic valve = blue arrow; anterior mitral valve leaflet = green arrow).

An urgent echocardiography was scheduled due to the clinical suspicion of infective endocarditis. The ejection fraction was normal, with severe aortic regurgitation, moderate mitral regurgitation and a bicuspid aortic valve as an incidental finding. Vegetations were discovered at the tips of the anterior and posterior aortic valves, measuring 1.1 cm × 0.6 cm and 1.6 cm × 0.8 cm, respectively. Another vegetation was discovered at the body of the anterior mitral valve leaflet, measuring 0.5 cm × 0.8 cm (Fig. 2).

He was diagnosed with infective endocarditis, which was complicated by septic embolization to multiple sites, including the brain, mesentery, kidney and spleen. Three sets of blood cultures were taken from three different sites at different times, and he was started on empirical antimicrobial treatment with intravenous benzylpenicillin and gentamicin. His condition improved after antibiotics, as his fever and abdominal pain subsided.

**Figure 3:**
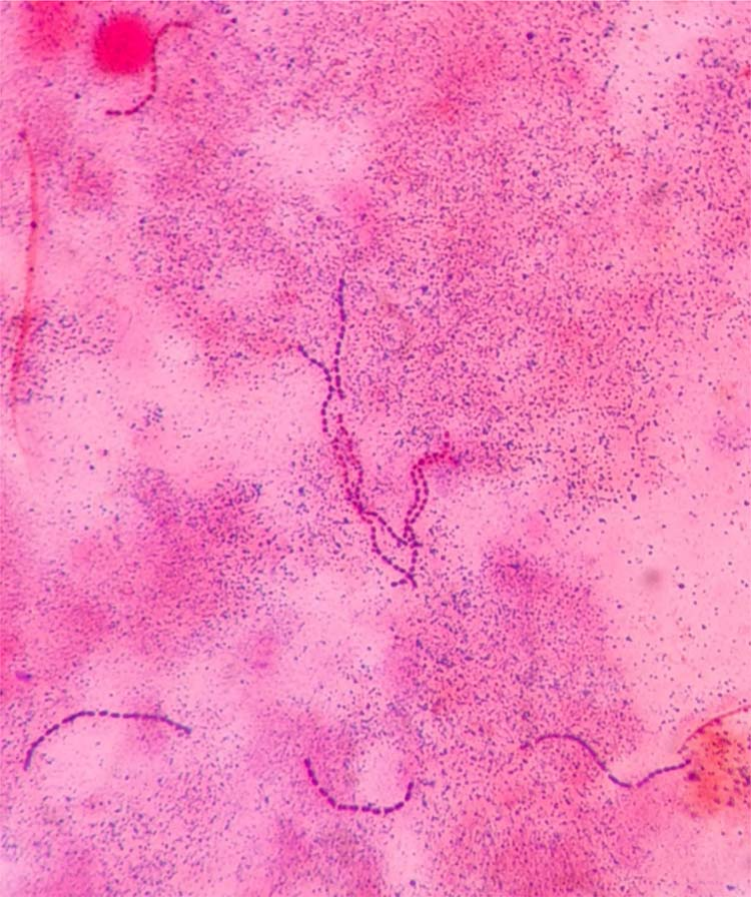
Gram stain of the blood culture showing gram-positive cocci grouped in chains.

After two days of incubation, *S. gordonii* was isolated from all blood cultures using the matrix-assisted laser desorption/ionization-time of flight mass spectrometry method (Fig. 3). The isolate was susceptible to penicillin, with a minimum inhibitory concentration (MIC) of 0.016 microgram/ml. He received benzylpenicillin 3 MU intravenously every 4 hours for a total of 6 weeks. He was referred to a cardiothoracic surgeon for surgical intervention due to the high risk of further septic embolization from multiple vegetations, the large size of vegetation (>1 cm), and previous systemic embolism. The patient was planned for surgical excision of the vegetations and valve reconstruction. He, however, decided to return to his home country for further treatment and valve surgery.

## DISCUSSION

VGS are catalase-negative, Gram-positive cocci arranged in chains on microscopic examination. The VGS can be classified into six major groups, namely *Streptococcus anginosus* group, *S. mitis* group, *S. sanguinis* group, *S. bovis* group, *Streptococcus salivarius* group, and *S. mutans* group. The *S. sanguinis* group is further divided into *S. sanguinis*, *Streptococcus parasanguinis*, and *S. gordonii* [4].

*S. gordonii* colonizes oral biofilms on tooth surfaces and is involved in the formation of dental plaques. Bacteria can be released from oral biofilms and enter the bloodstream via tooth brushing, tooth extraction or oral trauma, resulting in systemic infection. *S. gordonii* bacteremia can cause platelet aggregation and hyperinflammatory state, which contributes to the pathogenesis of infective endocarditis [5]. Our patient had no recent dental procedures. Nonetheless, dental plaques were discovered during a physical examination, which could be the source of *S. gordonii*.

*S. gordonii* is a rare causative agent of infective endocarditis. Mosailova *et al.* reported a 31-year-old man with mitral valve infective endocarditis caused by *S. gordonii* [6]. Besides that, Komorovsky *et al.* reported a case of *S. gordonii*-related infective endocarditis in an 11-year-old girl with Barlow’s mitral valve disease in which *S. gordonii* was cultured from the resected vegetation [7].

Systemic embolism is a devastating and potentially fatal complication of infective endocarditis that occurs in up to 50% of cases. In patients with infective endocarditis, this is usually caused by fragmentation of vegetation or cardiac tissue [8]. Millaire *et al.* reported that nearly half of patients with infective endocarditis developed systemic embolic complications, with the central nervous system being the most common, followed by the spleen, kidney, lung, liver, bone and joint, iliac artery, and mesenteric artery [9].

Infective endocarditis caused by highly penicillin-susceptible VGS, including *S. gordonii*, can be treated with parenteral penicillin or ceftriaxone. Gentamicin should be added for the first 2 weeks of penicillin treatment in patients with relatively penicillin-resistant VGS infective endocarditis. Early surgical intervention, in addition to targeted antimicrobial therapy, has been shown to reduce mortality and embolic events by lowering the risk of systemic embolism in patients with large vegetations [10].

In conclusion, infective endocarditis should be considered in young patients who have stroke-like symptoms. *S. gordonii* is a rare cause of infective endocarditis and is associated with dental plaques. Multiple, large-sized vegetations can predispose to systemic emboli, which must be treated promptly with antimicrobial therapy and early surgical intervention.

Abbreviations
VGS—Viridans group streptococci
CT—computed tomography
MALDI-TOF—matrix-assisted laser desorption/ionization-time of flight
MIC—minimum inhibitory concentration

## CONFLICT OF INTEREST STATEMENT

None declared.

## ETHICAL APPROVAL

Not required.

## CONSENT

Written informed consent was obtained from the patient.

## GUARANTOR

Edmund LC Ong is the guarantor.
